# A novel Mcl1 variant inhibits apoptosis via increased Bim sequestration

**DOI:** 10.18632/oncotarget.1147

**Published:** 2013-07-15

**Authors:** Judith Hagenbuchner, Ursula Kiechl-Kohlendorfer, Petra Obexer, Michael J. Ausserlechner

**Affiliations:** ^1^ Department of Pediatrics II, Medical University Innsbruck, Austria; ^2^ Department of Pediatrics I, Medical University Innsbruck, Austria; ^3^ Tyrolean Cancer Research Institute, Innsbruck, Austria

**Keywords:** Mcl1L, apoptosis, BCL2 proteins, mRNA variant

## Abstract

Members of the Bcl-2 protein family are frequently deregulated in tumors as they critically control cell death induction in mammalian cells. Alterations of these proteins may cause resistance to chemotherapy-induced cell death and immune responses. By serendipity we cloned a variant of the anti-apoptotic Bcl2-family member Myeloid cell leukemia-1 (Mcl1) from human neuroblastoma and leukemia cells. This Mcl1L variant lacks a 45 bp sequence that codes for 15 highly conserved amino acids ranging from Gly158 to Asp172. This region is part of the so called PEST-sequence of Mcl1L and contains two phosphorylation sites (Ser159 and Thr163) that regulate Mcl1L stability. A caspase 3/caspase 8 cleavage site at Asp157 which has been reported to be critical for death-receptor-induced apoptosis and for the conversion of Mcl1L into a pro-apoptotic protein is also missing in this novel variant. Importantly, Mcl1L_delGly158-Asp172_ bound significantly more pro-apoptotic Bim compared to Mcl1L and showed increased anti-proliferative and anti-apoptotic activity compared to Mcl1L during death receptor-induced cell death. This suggests that this novel Mcl1L variant efficiently protects tumor cells against extrinsic death signalling and therefore may provide a survival advantage for highly aggressive tumors.

## INTRODUCTION

Mcl1 was originally identified in differentiating myeloid cells [1] and has unique structural features among the members of the anti-apoptotic BCL2 family. The C-terminal part (aa 170-300) of Mcl1 shares structural similarities with other anti-apoptotic BCL2 family members, like BclxL. The N-terminal part, however, lacks the characteristic BH4 domain and instead contains two highly conserved proline, glutamic acid, serine and threonine-rich PEST sequences [2]. The second PEST sequence includes also two caspase cleavage sites (Asp127, Asp157) and several phosphorylation sites that are involved in regulating Mcl1 function and stability [3-6]. Mcl1L expression is controlled by various transcriptional, post-transcriptional and post-translational pathways downstream of growth factor- and cytokine signaling [7-9]. Thr163 is the main phosphorylation site in Mcl1 regulating stability, function and association with pro-apoptotic BH3-only proteins. ERK-mediated phosphorylation at Thr163 and Thr92 increases Mcl1-stability by binding to Pin-1 [10] as well as its anti-apoptotic function. Stress-induced phosphorylation on Ser121 and Thr163 inactivates Mcl1 pro-survival function [11, 12] and combined phosphorylation at Thr163 and Ser159 by JNK and GSK3β destabilizes Mcl1 as well as reduces its interaction with pro-apoptotic Bim [13]. Beside phosphorylation the interaction with distinct BH3-only proteins also coordinates Mcl1 expression, function and stability. Mcl1 can bind and thereby inactivate pro-apoptotic Bak [14, 15] but this complex can be either disrupted via extrinsic death signaling by tBid or intrinsically by induction of PMAIP1/Noxa, leading to proteasomal degradation of Mcl1 and apoptosis induction via Bak-oligomerisation [15-18]. Binding and inactivation of Bim and Puma increases Mcl1 levels, protects Mcl1 from degradation and acts anti-apoptotic by sequestration of Bim [17, 19, 20]. Cleavage of Mcl1 by caspase-3 or 8 during TRAIL-induced apoptosis, however, releases sequestered Bim and causes apoptosis via activation of Bax. The cleavage and inactivation of Mcl1L by caspases represents a second, Bid-independent linkage between extrinsic and intrinsic death pathway [16, 21]. Proteasomal degradation of Mcl1 is controlled by different E3-ubiquitin ligases. The most prominent is MULE, which is thought to regulate the constitutive turnover of Mcl1L by binding via its BH3-domain to the hydrophobic pocket of Mcl1L [6]. Two additional E3-ligases have been identified that regulate Mcl1L ubiquitination: During apoptosis execution and triggered by GSK3β-induced phosphorylation of Mcl1L the E3-ligases SCF^FBW7^ and β-TRCP regulate Mcl1 degradation [22, 23]. The activity of these E3-ligases is counteracted by the de-ubiquitinase USP9X which removes Lys48-linked polyubiquitine-chains and thereby stabilizes Mcl1L and increases its anti-apoptotic function [18, 24].

Beside anti-apoptotic full-length Mcl1L, there is evidence for several pro-apoptotic Mcl1 variants. Pro-apoptotic variants are generated either by cleavage of Mcl1 by caspase-3 or 8 [25, 26] or by alternative splicing. Loss of exon 2 results in the translation of Mcl1s (short, 271aa), a splice variant which only contains the BH3-domain and inactivates Mcl1L thereby acting like a pro-apoptotic BH3-only protein [27, 28]. Such a pro-apoptotic variant is also known for BclxL, where alternative splicing generates BclxS [29]. Splicing in exon1, at a non-canonical splice site, leads to Mcl1ES (extra short, 197 aa), which lacks the PEST sequence but binds Mcl1L. This variant does not sequester Bax or Bak and thereby acts pro-apoptotic [30]. In the present study we characterized a novel Mcl1 splice variant, which was cloned from human neuroblastoma and leukemia cells. As this variant lacks important regulatory parts of the PEST sequence we hypothesized that tumor cells expressing this Mcl1 variant may gain survival advantages and escape certain death stimuli.

## RESULTS

### Cloning of a novel variant of human Mcl1 in human cancer cells

**Figure 1:**
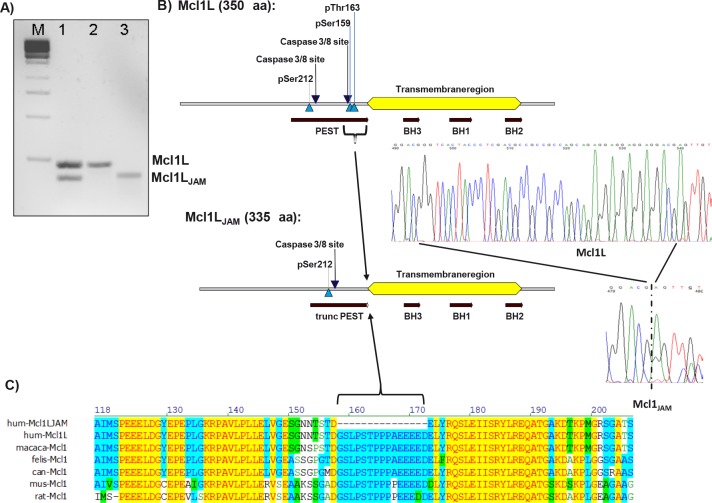
Cloning of a novel mRNA variant of Mcl1L Nested PCR of cDNA from C7H2 leukemia cells (lane 1). Expression vectors for either Mcl1L or Mcl1L_JAM_ were used as positive controls (lane 2 and 3). Mcl1L_JAM_ lacks 45 bp within the coding region of Mcl1L mRNA which codes for the amino acids Gly158 to Asp172. These 15 amino acids are part of the PEST sequence of MCL1L (b) which is highly conserved within different mammalian species (c).

In the course of PCR analyses of Mcl1 mRNA variants in human neuroblastoma cells we amplified an mRNA species that carries a 45 bp deletion in the Mcl1L coding region from SH-EP neuroblastoma cells. For simplicity we termed this Mcl1L_del158-172_ variant Mcl1_JAM_ (*J*ust *A*nother *M*cl1). PCR-products corresponding to the full length and the Mcl1L_JAM_ variant were detected also in a CEM leukemia cell line (Fig. 1A). The shortened Mcl1 variant lacks a 15 amino acids region ranging from Gly158 to Asp172 that contains a caspase 3/8 cleavage site and the two important regulatory amino acids Ser159 and Thr163 (Fig. 1B). This part of Mcl1 is highly conserved within mammals (Fig. 1C). The phosphorylation of Mcl1L on Ser159 and Thr163 by GSK3β, ERK or JNK is critical for Mcl1 stability and its interaction with pro-apoptotic BH3-only proteins [13].

The region Gly158 to Asp172 is the most proline- and glutamate-rich part of the PEST region which acts as a signal sequence for proteasomal degradation and determines the short protein half-life of Mcl1. As the functional consequences of lack of this region are unclear, we next investigated how the function of Mcl1L_JAM_ differs from Mcl1L and if this novel variant affects the physiology and death resistance of human neuroblastoma cells.

### Mcl1L_JAM_ is an unstable variant that enhances the anti-proliferative effect of Mcl1L

**Figure 2:**
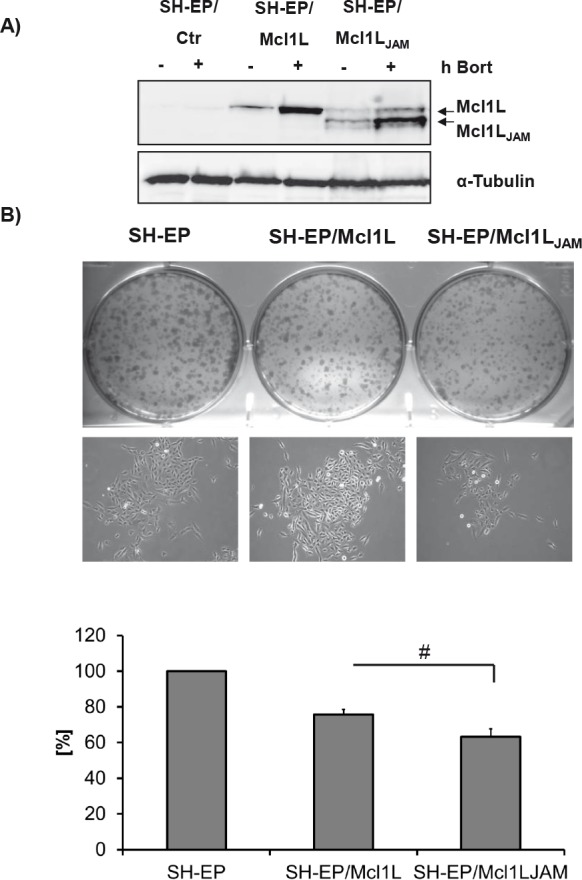
The Mcl1L mRNA variant Mcl1L_JAM_ exerts anti-proliferative effect SH-EP/Ctr, SH-EP/Mcl1L and SH-EP/Mcl1L_JAM_ cells were treated for four hours with 200 nM bortezomib (bort). Cell lysates were subjected to immune blot to verify ectopic expression of Mcl1L and Mcl1L_JAM_. α-Tubulin served as a loading control (a). Colony formation was analysed by crystal violet staining of 2×10^3^ SH-EP/Ctr, SH-EP/Mcl1L and SH-EP/Mcl1L_JAM_ cells after 7 days cultivation (b, upper and lower panel). Bright field analyses of single colonies were performed in an Axiovert200M microscope (b, middle panel). Shown is the mean of four independent experiments. Statistical analysis was performed using students t-test (* P < 0.05).

Besides playing a key role in the regulation of mitochondrial cell death Mcl1L and the recently described proteolytic fragment snMcl1 were also implicated in the regulation of cell cycle progression. Mcl1L binds to PCNA in the nucleus and thereby inhibits proliferation, whereas snMcl1 reduces CDK1 activity [31, 32]. To assess whether Mcl1L_JAM_ differs in its anti-proliferative activity from full length Mcl1L SH-EP cells were infected with a retrovirus vector containing either the coding sequence for Mcl1L full-length or the mRNA variant Mcl1L_JAM_. The expression of the smaller variant was verified by immunoblot in presence or absence of the proteasome inhibitor Bortezomib. Similar to Mcl1L Bortezomib treatment led to accumulation of the variant Mcl1L_JAM_ suggesting that despite its truncated PEST sequence, Mcl1_JAM_ is still degraded via the proteasome (Fig. 2A). Ectopic Mcl1L expression reduced the colony forming capacity of SH-EP neuroblastoma cells to 74.4% compared to mock-infected controls (100%) but did not influence the colony size (Fig. 2B). Ectopic Mcl1L_JAM_ expression, however, further reduced the number of colonies to 66% compared to SH-EP/Ctr cells and also interestingly reduced colony size (Fig. 2B). This suggests that Mcl1L_JAM_ reduces the ability of single cells to form colonies and, in contrast to Mcl1L, also reduces colony size suggesting a more pronounced anti-proliferative effect of this variant.

### Lack of Gly158 to Asp172 reduces protein stability

**Figure 3:**
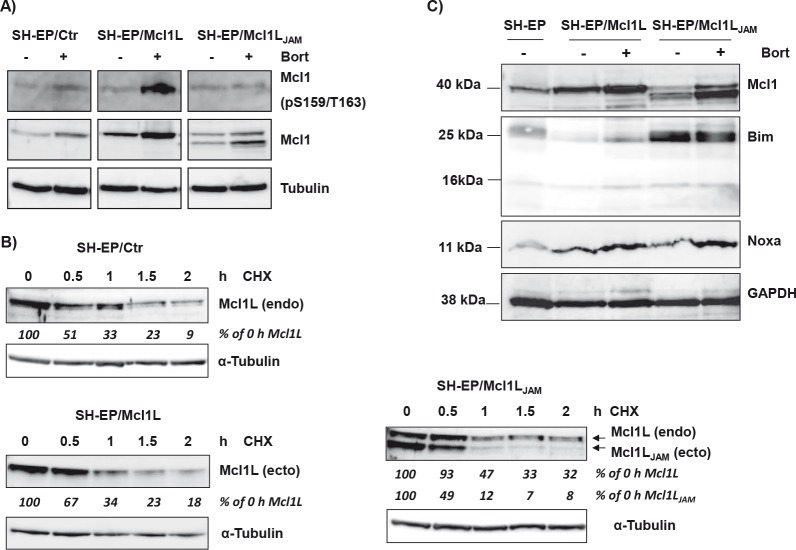
Mcl1L_JAM_ rescues Mcl1L from degradation and cooperates with Bim SH-EP/Ctr, SH-EP/Mcl1L and SH-EP/ Mcl1L_JAM_ cell lysates were analysed for phosphorylated Mcl1 (Ser159/Thr163) and Mcl1 after treatment with bortezomib for four hours (a). α-Tubulin served as loading control. Protein stability was analysed by treating SH-EP/Ctr, SH-EP/Mcl1L or SH-EP/Mcl1L_JAM_ cells with 10 μg/ml CHX for the times indicated (b). α-Tubulin served as loading control. SH-EP/Ctr, SH-EP/Mcl1L and SH-EP/Mcl1L_JAM_ cells were treated for four hours with 200 nM bortezomib. Cell lysates were analysed for the expression of Noxa and Bim. GAPDH served as loading control (c).

Mcl1L function, interactions and degradation are critically regulated by phosphorylation of Ser159 and Thr163 within the PEST region (reviewed in [33]). As Mcl1L_JAM_ exerted enhanced anti-proliferative activity compared to Mcl1L and lacks the region containing several proline and glutamate amino acids and these two important phosphorylation sites we hypothesized that Mcl1L_JAM_ may have an altered half-life compared to Mcl1L. We therefore analysed phosphorylation at Ser159 and Thr163 during bortezomib treatment. In cells ectopically expressing Mcl1L the inhibition of proteasome-mediated protein degradation resulted in significant accumulation of Mcl1L phosphorylated at Ser159/Thr163. This implies that in control cells Ser159/Thr163 are phosphorylated and Mcl1L is subject to permanent turnover. Interestingly, the Bortezomib-treatment of neuroblastoma cells overexpressing Mcl1L_JAM_ mainly resulted in accumulation of this novel variant, whereas the endogenous Mcl1L remained unphosphorylated and only slightly accumulated. This suggests that despite truncation of the PEST sequence, Mcl1L_JAM_ competes with endogenous Mcl1L for binding partners that mediate the degradation of Mcl1 (Fig. 3A). Next we analysed possible differences between protein half-life of Mcl1L and Mcl1L_JAM_ by treating the Mcl1L or Mcl1L_JAM_-expressing cells with 10 μg/ml cycloheximide (CHX) for up to 3 hours. In SH-EP/Ctr cells endogenous Mcl1L steady state expression is already significantly lowered within 30 minutes of CHX treatment to about 50 % (Fig. 3B, upper panel). Similar results were obtained for cells expressing ectopic Mcl1L with about 67% of Mcl1L being present after 30 min. The higher amounts of Mcl1L in the overexpression system compared to endogenous Mcl1L suggests that the CHX block of translation was not 100% complete (Fig. 3B, lower panel). Despite the shortened PEST sequence overexpressed Mcl1L_JAM_ had a slightly lower stability than overexpressed Mcl1L (49% versus 67% after 30 min) with a rapid decay after 60 min (12% versus 34%) and 90 min (7% versus 23%). However, the expression of Mcl1L_JAM_ stabilized endogenous Mcl1L and significantly increased the half-life of endogenous Mcl1L to 93% after 30 min and still 47% after one hour (Fig. 3B, right panel). This suggests that the variant Mcl1L_JAM_ has a short half-life but protects Mcl1L from proteasomal degradation causing its stabilization and may therefore directly and/or indirectly affect apoptosis sensitivity.

### Lack of Gly158 to Asp172 increases steady state expression of pro-apoptotic Bim

**Figure 4:**
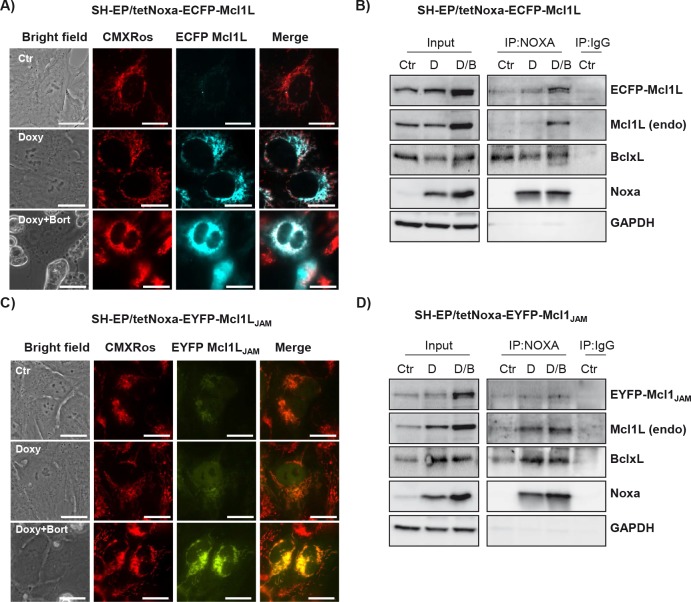
Mcl1L, but not Mcl1LJAM, inactivates Noxa SH-EP/tetNoxa-ECFP-Mcl1L or SH-EP/tetNoxa-EYFP- Mcl1L_JAM_ cells were treated with 200 ng/ml dox(D), or with a combination of 200 ng/ml doxy and 200 nM bortezomib (D/B) for 4 hours. Cells were either subjected to live-cell microscopy (a, c) or co-immunoprecipiation (b, d). For live cell imaging, mitochondria were stained with 300 nM CMXRos (bar=10 μm). After precipitation of Noxa, immunoblots were performed against Noxa, Mcl1, GFP, BclxL or GAPDH. Mouse-serum was used as precipitation control (IgG) (b, d).

Phosphorylation at Ser159 and Thr163 not only induces destabilization of Mcl1L but was also reported to decrease the ability of Mcl1L to bind and inactivate pro-apoptotic protein Bim [13]. We therefore next studied how deletion of the region Gly158 to Asp172 in Mcl1L might affect expression and interaction with pro-apoptotic Mcl1L-binding partners. Overexpression of Mcl1L is associated with elevated steady state protein levels of Noxa, which can be further increased by Bortezomib-treatment (Fig. 3C). This suggests that increased levels of anti-apoptotic Mcl1L sequester higher amounts of the pro-apoptotic binding partner Noxa and thereby allow a cell to cope with increased cellular levels of cell death inducers. Interestingly SH-EP/Mcl1L_JAM_ cells did not contain elevated Noxa-levels, although Bortezomib-treatment increased Noxa steady state levels similar to SH-EP/Mcl1L cells (Fig. 3C). Mcl1L_JAM_ overexpression instead clearly increased basal Bim levels compared to SH-EP/Mcl1L or SH-EP/Ctr cells, suggesting that Mcl1L_JAM_ may efficiently prevent the death-inducing effect of Bim, possibly by sequestration. These Mcl1L_JAM_ -specific changes in the steady state expression of Noxa and Bim are also observed, when Mcl1L or Mcl1L_JAM_ are expressed in leukaemia cell lines (Supplemental figure S2), suggesting that the specific accumulation of Noxa or Bim depends on structural characteristics of Mcl1L and Mcl1L_JAM_, respectively. To study whether the increased expression levels correlate with an increased interaction of these proteins, we generated cells with tetracycline-inducible expression of Noxa or Bim and constitutively expressed ECFP-tagged Mcl1L (ECFP-Mcl1L) or EYFP-tagged Mcl1L_JAM_ (EYFP- Mcl1L_JAM_). Treatment of SH-EP/tetNoxa-ECFP-Mcl1L cells with doxycyline (doxy) induces Noxa expression (Fig. 4B, lane 2) but also increased the ECFP-Mcl1L signal (Fig. 4A) and ECFP-Mcl1L steady state expression as measured by immunoblot (Fig. 4B). This suggests that increased levels of Noxa lead to the accumulation of more stable Noxa/Mcl1L complex in neuroblastoma cells [34]. A possible explanation for this stabilization might be that Noxa binds into the BH3-domain of Mcl1L displaces the ubiquitine-ligase MULE from Mcl1L and thereby reduces Mcl1L turn over [35, 36].

In contrast, doxycycline-induced expression of Noxa in SH-EP/tetNoxa-EYFP- Mcl1L_JAM_ cells did not increase the fluorescence intensity or protein steady state levels of ECFP- Mcl1L_JAM_ although Noxa still elevated protein levels of endogenous Mcl1L (Fig. 4C). Co-immunoprecipiation experiments of Noxa revealed that in SH-EP/tetNoxa-ECFP-Mcl1L cells endogenous Mcl1L and ECFP-Mcl1L precipitated with Noxa, with increased amounts when Mcl1 degradation is blocked by proteasome inhibition (Fig. 4B). Since BclxL is also an interaction partner of Noxa in neuroblastoma cells [34], we also analysed BclxL in Noxa-precipitates and found low amounts of BclxL bound to Noxa in Mcl1L-overexpressing cells (Fig. 4B). This suggests a basal interaction of BclxL and Noxa and if Noxa expression is further elevated part of Noxa is sequestered by Mcl1L. In contrast, in SH-EP/tetNoxa-EYFP-Mcl1L_JAM_ cells only small amounts of EYFP-Mcl1L_JAM_ precipitated with Noxa, whereas endogenous Mcl1L binds to Noxa in the same manner as in ECFP-Mcl1L-overexpressing cells. In these cells, however, significantly increased amounts of BclxL co-purified with Noxa (Fig. 4D). This suggests that Noxa interacts with BclxL and Mcl1L at higher affinity than with Mcl1L_JAM_.

**Figure 5:**
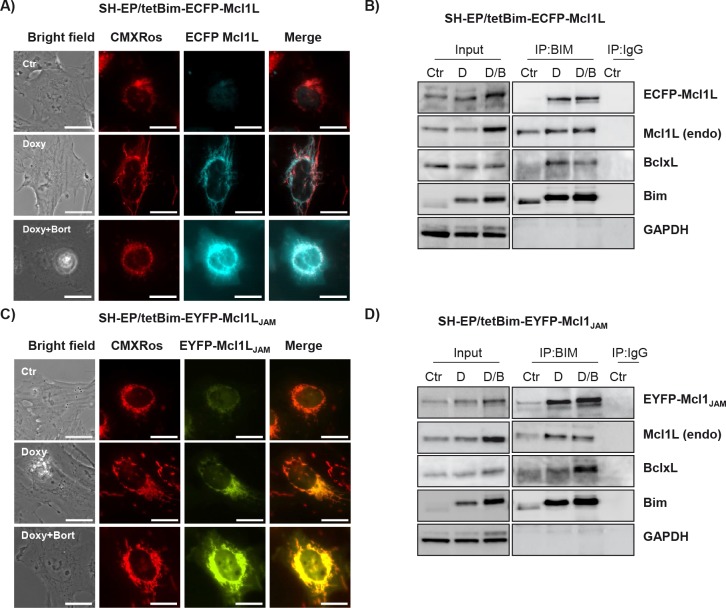
Mcl1LJAM interacts preferentially with Bim SH-EP/tetBim-ECFP-Mcl1L or SH-EP/tetBim-EYFP- Mcl1L_JAM_ cells were treated with 200 ng/ml doxy(D) to induce Bim, or with a combination of 200 ng/ml doxy and 200 nM bortezomib (D/B) for 4 hours. For live-cell imaging, cells were analysed after CMXRos (300 nM) staining of mitochondria (a). Bar represents 10 μm. After precipitation of Bim, immunoblots were performed against Bim, Mcl1, GFP, BclxL or GAPDH. Rabbit-serum was used as precipitation control (IgG) (b, d).

In SH-EP/tetBim-EYFP-Mcl1L_JAM_ and SH-EP/tetBim-ECFP-Mcl1L Bim-induction (doxy-treatment) increased steady state levels of Mcl1L_JAM_ and Mcl1L which is in line with results from Wuillème-Toumi et al, who found that Bim and Mcl1L protect each other from degradation [20]. By co-immunoprecipitation experiments we found that Bim interacted with endogenous Mcl1L, ECFP-Mcl1L and BclxL which similar affinity, suggesting that increased Bim amounts equally distribute between Mcl1L and BclxL (Fig. 5B). In SH-EP/tetBim-EYFP-Mcl1L_JAM_ cells, doxy-induced Bim precipitated EYFP-Mcl1L_JAM_ and to a lesser extend endogenous Mcl1L. Co-purification of BclxL was only observed in cells treated with both, doxy and bortezomib (Fig. 5D). This combined effect may be due to the fact that bortezomib further induces Noxa, which partially sequesters Mcl1L and BclxL in neuroblastoma cells [34]. The combined data suggest that Mcl1L_JAM_ efficiently binds and inactivates Bim and thereby changes the interaction of this pro-apoptotic protein with other Bcl2 proteins. In the next step we therefore analysed whether Mcl1L_JAM_ expression changes the sensitivity to distinct apoptosis-stimuli of extrinsic and intrinsic apoptosis signalling.

### Increased expression of Mcl1L_JAM_ protects neuroblastoma cells against death receptor-mediated apoptotic cell death

**Figure 6:**
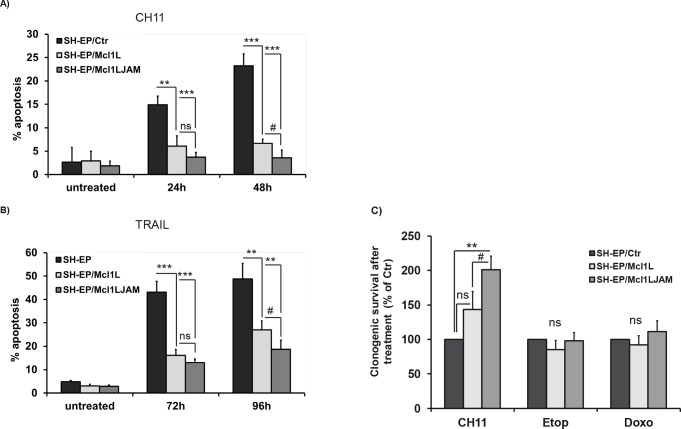
Mcl1L_JAM_ protects against extrinsic death signalling SH-EP/Ctr, SH-EP-Mcl1L and SH-EP/Mcl1L_JAM_ cells were treated with either 100 ng/ml FAS-activating CH11 antibody for 0, 24 and 48 hours (a) or 0.1μg/ml recombinant TRAIL for 72 and 96 hours (b) and subjected to PI-FACS analyses. Shown is the mean of three independent experiments. 4×10^4^ SH-EP/Ctr, SH-EP/Mcl1L and SH-EP/Mcl1L_JAM_ cells were seeded into 6well plates and treated with or without 100 ng/ml CH11, 20 μg/ml etoposide or 0.25 μg/ml doxorubicin for 72 hours. Surviving cell colonies were fixed and stained with crystal violet/methanol and de-stained with SDS/ethanol. Supernatants were measured photometrically and treated SH-EP/Ctr cells were set as 100%. Shown is the mean of four independent experiments (c). Statistical analysis was performed using students t-test (** P < 0.01, *** P < 0.001 difference between Ctr and Mcl1L or Mcl1L_JAM_, # P < 0.05.difference between Mcl1L and Mcl1L_JAM_).

SH-EP/Ctr, SH-EP/Mcl1L or SH-EP/Mcl1L_JAM_ cells were treated either with the chemotherapeutics etoposide and doxorubicin that are expected to mainly trigger the intrinsic apoptotic pathway or with FASL/CH11 or TRAIL to induce apoptosis via death receptors. Interestingly, both Mcl1L and Mcl1L_JAM_ only inhibited FASL- and TRAIL-induced apoptosis (Fig. 6AB), whereas they failed to rescue neuroblastoma and leukemia cells from etoposide or doxorubicin-induced cell death (Figure 6C and Supplemental figure 1AB). Mcl1L_JAM_ expression significantly reduced cell death from 48.8% to 18.8% after TRAIL treatment and from 23.3% to 3.6% after CH11 treatment. Mcl1L_JAM_ thereby induced significantly higher death resistance than Mcl1L overexpression after 48 hours (P < 0.05). To study long-term survival after treatment we performed clonogenic survival assays which demonstrated that survival of neuroblastoma cells after FAS-receptor activation by CH11 antibody was significantly increased in Mcl1L_JAM_ -expressing cells (201%, P < 0.01) compared to Mcl1L-expressing cells (143%) and SH-EP/Ctr cells (Fig. 6C). However, no significant differences were detectable in etoposide or doxorubicin treated cells (Fig. 6C). Taken together, these data suggest that deletion of the sequence Gly158 to Asp172 in Mcl1L_JAM_ confers increased resistance to death receptor-induced apoptosis and thereby may provide a survival advantage for tumor cells.

## DISCUSSION

In the present study we functionally analysed the novel Mcl1L mRNA variant Mcl1L_delGly158-Asp172_ which we named Mcl1L_JAM_. This variant was cloned by serendipity from human neuroblastoma and leukemia cells and lacks a 15 amino acids region (Gly158-Asp172) including a caspase 3/8 cleavage site and two regulatory amino acids (Ser159, Thr163). Both, Mcl1L and Mcl1L_JAM_ accumulate after proteasome inhibition by bortezomib treatment (Fig. 3A), suggesting that the lack of the sequence Gly158 to Asp172, although being a critical part of the PEST sequence does not prevent proteasomal degradation. Three ubiquitin ligases have been shown to control degradation of Mcl1: β-TrCP and SCF^FBW7^ both require phosphorylation at Ser159 and Thr163 for Mcl1 recognition/degradation via the proteasome, which makes it unlikely that these two E3-ligases also regulate the turnover of Mcl1L_JAM_. In contrast the ubiquitin ligase MULE/ARF-BP1 which specifically binds to Mcl1L via its BH3-domain might be a possible candidate, especially as protein levels of Mcl1L and Mcl1L_JAM_ were affected by conditional expression of BH3-only proteins Noxa and Bim, respectively [6, 22, 23]. Bortezomib-treatment not only caused Mcl1L accumulation, but Mcl1L was also strongly phosphorylated at Ser159 and Thr163 (Fig. 3A), suggesting that the upstream kinase cascades that modulate Mcl1L activity and stability via Ser159/Thr163 phosphorylation are active in these neuroblastoma cells.

The loss of the phosphorylation site at Thr163 suggests altered stability and function of Mcl1L_JAM_ compared to full length Mcl1L since phosphorylation at Thr163 represents the main regulatory phosphorylation site in Mcl1L [33]. Combined phosphorylation at Thr163 und Thr92 through ERK-1 increases Mcl1 stability through association with Pin-1 [10] whereas phosphorylation at Thr163 together with Ser159 by JNK and GSK-3β decreases Mcl1 stability as well as binding to Bim [12, 13]. Actually Mcl1L_JAM_ even showed reduced stability during CHX treatment (Fig. 3B) compared to Mcl1L. Ectopic Mcl1L_JAM_ disappeared almost completely after 1 hour (12%) whereas ectopic Mcl1L showed a similar decay as endogenous Mcl1L during CHX treatment. This is in line with a recent report from Thomas et al, who demonstrated that mutation at Ser162 reduces the stability of Mcl1 [37]. Surprisingly, ectopic expression of Mcl1L_JAM_ enhances the half-life of endogenous Mcl1L suggesting that both proteins compete for the same binding partners and Mcl1L_JAM_ is preferentially targeted for degradation and therefore more instable than the wild type. Since no ubiquitin residues are affected by the deletion in Mcl1_JAM_ (Lys5, 40, 136, 194 and 197) [6] we hypothesize that the degradation may be caused by changes in the C-terminus of Mcl1L_JAM_, which might affect binding to BH3-only proteins, for example Puma which was shown to protect Mcl1L from MULE induced degradation by binding to its BH1 domain, [36] or Bim-interaction [20]. So expression of the shortened variant may protect wild type Mcl1L from its degradation, which is likely to provide a survival advantage to Mcl1L_JAM_ -expressing cancer cells. We also observed this enhanced stability of Mcl1L when coexpressed with Mcl1L_JAM_ after treatment with CH11 or TRAIL. Mcl1L_JAM_ was degraded after CH11 and TRAIL treatment but delayed phosphorlation and degradation of Mcl1L (Supplemental figure S3). The elevated apoptosis inhibitory function of Mcl1L_JAM_ was limited to extrinsic death signaling, as both Mcl1L and Mcl1L_JAM_ failed to reduce cell death induced by etoposide and doxorubicin treatment (Fig. 6C and supplemental figure S1). In SH-EP neuroblastoma cells efficient death receptor signalling requires involvement of mitochondria [38]. Our data suggest that the Mcl1L_JAM_ variant specifically interferes with the connection between extrinsic and intrinsic death signaling. Once caspase-8 is activated, it may directly cleave Mcl1L at Asp157 leading to its conversion into a pro-apoptotic Bcl2-protein [39] and to changes in the sequestration of pro-apoptotic Bcl2 proteins [21]. Since Mcl1L_JAM_ lacks Gly158, it may be protected from cleavage. Additionally caspase-8 cleaves and activates Bid, a strong BH3-only protein that neutralizes Mcl1L. Since Mcl1L_JAM_ showed increased affinity to Bim compared to Mcl1L (Fig. 4 and Fig. 5), tBid may not be able to disrupt Mcl1_JAM_/Bim complexes, resulting in prolonged inactivation of Bim in Mcl1_JAM_-expressing cells. Deletion of the entire Mcl1L N-terminus changes the C-terminal part in a way that an increased binding with Bim is observed [21]. Interestingly, the same is also true for the short 15 amino acid deletion present in Mcl1L_JAM_ (Fig. 5B) suggesting conformational changes in the C-terminal part of this protein variant that result in altered hydrophobic BH3 binding [2]. Consistent with this hypothesis Mcl1L_JAM_ completely failed to sequestrate increased cellular Noxa amounts upon tetracycline-regulated Noxa induction, (Fig. 4D). Instead, Noxa mainly interacted with endogenous BclxL and Mcl1L (Fig 4B). This suggests that Noxa binds with low affinity to Mcl1L_JAM_ in neuroblastoma cells, whereas Bim is efficiently sequestrated by this variant.

A current study by Thomas et al identified Ser162 as key phosphorylation site regulating Mcl1 cellular localization. If this site is mutated Mcl1L almost exclusively localizes to the nucleus [37]. These changes in Mcl1 localization also lead to reduced stability and less apoptosis protection against mitochondria-induced cell death. Life cell imaging analyses of SH-EP cells infected with the EYFP-Mcl1L_JAM_ construct uncovered a partial nuclear localization after stabilization of the protein with bortezomib (Supplemental figure S4, upper panel). Subcellular fragmentation experiments also detected large amounts of EYFP-tagged Mcl1jh in the nuclear extracts, also in untreated cells, whereas a small amount of endogenous Mcl1L was also detected in the nucleus after proteasome-inhibition (Supplemental figure S4, lower panel). In line with this report, Mcl1L_JAM_ was not able to inhibit mitochondrial cell death, but increases resistance to death receptor induced apoptosis (Fig 6ABC). This suggests that loss of this short peptide sequence in Mcl1 significantly affects stability, interaction with BH3-only proteins and also death sensitivity to distinct apoptotic signals. Expression of this Mcl1 variant may therefore represent an adaption of tumor cells to avoid extrinsic death signaling and may thereby serve as a diagnostic and/or therapeutic gene in neuroblastoma and other malignancies.

## MATERIALS AND METHODS

### Cell lines, culture conditions, and reagents

The lines SH-EP [40], CEM-C7H2 [41] and Phoenix™ packaging cells [42] were cultured in RPMI1640 (Lonza, Basel, Switzerland) containing 10% fetal calf serum, 100u/ml penicillin, 100μg/ml streptomycin (PAA, Pasching, Austria) and 2mM L-glutamine (Lonza, Basel, Switzerland) at 5% CO_2_. All cultures were routinely tested for mycoplasma contamination using the Venor^R^ GeM-mycoplasma detection kit (Minerva Biolabs, Berlin, Germany).

### Retroviral vectors and production of retroviruses

pLIB-MCS2-iresPuro, pLIB-MCL1-iresPuro pLIB-rtTA-M2-iresTRSID-iresPuro, pQ-tetCMV-Bim-SV40Neo, pQ-tetCMV-Noxa-SV40Neo, pQ-tetH1-SV40-Puro have been described before [34, 43, 44]. The coding sequence for Mcl1L_JAM_ was amplified from human cDNA and inserted into the BamH1-EcoRI sites of pLIB-MCS2-iresPuro. For live-cell imaging, the coding sequence of ECFP or EYFP were amplified from pECFP-C1 and pEYFP-C1 (Clontech Laboratories Inc, Mountain View, CA, USA) and inserted into the EcoR1 site of pLIB-Mcl1L-iresPuro (resulting in pLIB-ECFP-Mcl1L-iresPuro) or pLIB-Mcl1L_JAM_-iresPuro (resulting in pLIB-EYFP-Mcl1L_JAM_-iresPuro). The production of retrovirus supernatants was described previously [43]. The retroviral supernatants were used to generate SHEP/Ctr, SH-EP/Mcl1L, SH-EP/Mcl1L_JAM_, SH-EP/ECFP-Mcl1L or SH-EP/EYFP-Mcl1L_JAM_ cells. The tetracyclin-regulated cell lines SH-EP/tetNoxa and SH-EP/tetBim have been described previously [34, 44]. These cells lines were further infected with supernatants containing either pLIB-ECFP-Mcl1L-iresPuro or pLIB-EYFP-Mcl1L_JAM_-iresPuro, generating SH-EP/tetNoxa-ECFP-Mcl1L, SH-EP/tetNoxa-EYFP-Mcl1L_JAM_, SH-EP/tetBim-EYFP- Mcl1L_JAM_ and SH-EP/tetBim-ECFP-Mcl1L cells.

### Detection of Mcl1L_JAM_ by PCR

Mcl1L_JAM_ expression was analysed via RT-PCR. For PCR detection the coding sequence of MCL1 was amplified from cDNA and a nested PCR was performed using 5' AAGAGGAGCTGGACGGGTAC and 3' TGGCTTTGTGTCCTTGGC which amplifies part of the PEST region. PCR products were analysed on 2% agarose gels.

### Immunoprecipitation and Immunoblotting

For immuoprecipitation 1×10^7^ cells were lysed in PBS containing 1% IGEPAL, phosphatase- and protease-inhibitors. For immunoprecipitation 1 μg of anti-rabbit Bim antibody (Cell Signaling Technology Inc., Boston, MA, USA), 2.5 μg of anti-mouse Noxa (Abcam, Cambridge, UK), or mouse or rabbit immunoglobulin, as a negative control, were covalently coupled to Tachisorb™ Immunoadsorbent (Calbiochem, Nottingham, UK) or Affi-Prep Protein A Support (BioRad Laboratories, Munich, Germany) using dimethylpimelidate dihydrochloride/Borax buffer. Antibody-bead complexes were added to 500 μg lysate and incubated at 4°C for 6 hours. Tachisorb™-/Protein A-immunocomplexes were washed four times in PBS/IGEPAL-buffer, resuspended in SDS-sample buffer and subjected to SDS-PAGE and blotting. Equal amounts of total-protein and cleared supernatants were loaded as controls. Immunoblot analysis was performed as previously described [45] using primary antibodies directed against human Bim and Mcl1 (BD-Pharmingen, USA, pMcl1(Ser159/Thr163) and BclxL (Cell Signaling Technology Inc., Boston, USA), Noxa (Alexis Biochemicals, San Diego, CA, USA), GFP (Sigma, Vienna, Austria) and GAPDH (Acris antibody GmbH, Herford, Germany). The membranes were then washed and incubated with horseradish-peroxidase-conjugated anti-mouse or anti-rabbit secondary antibodies (Amersham Biosciences, Buckinghamshire, UK). The blots were developed by enhanced chemiluminescence (GE-Healthcare, Vienna, Austria) and measured with an AutoChemi detection system. Densitometry analysis was performed using LabWorks software (UVP, Cambridge, UK).

### Colony forming assay (CFA)

To determine the ability of SH-EP cells to form colony units, 2×10^3^ cells were seeded into a 6well and cultured up to 7 days. For chemotherapeutic treatment 4×10^4^ cells were seeded into 6wells and treated for 72 hours with chemotherapeutic agents (CH11, etoposide, doxorubicin). Afterwards medium was removed and cells were fixed and stained with 0.2% crystal violet in 50% methanol. Cell density was measured photometrically after discoloration with 0.5% SDS in 50% ethanol. Untreated/ mock-infected cells were set as 100%.

### Flow cytometry analyses

Apoptosis was assessed by staining the cells with propidium-iodide (PI) using a CytomicsFC-500 Beckman Coulter as previously described [46]. In short: 2×10^5^ cells were harvested and resuspended in hypotonic PI solution for 2-4 hours at 4°C. Stained nuclei in the sub-G1 marker window were considered to represent apoptotic cells. Statistical analysis was performed using GraphPad Prism 4.0 software.

### Live cell fluorescence microscopy

For live cell analyses cells were grown on LabTek Chamber Slides™ (Nalge Nunc International, Rochester, NY, USA) coated with 0.1 mg/ml collagen. Images were collected with an Axiovert200M microscope with a 63x-oil objective (Zeiss, Vienna, Austria). Mitochondria staining was performed using 300 nM CMXRos (Invitrogen, Carlsbad, USA).

### Statistical analysis

Statistical significance of differences between controls and treated cells were calculated using unpaired t-test. All statistical analyses were performed using Graph Pad Prism 4.0 software.

## Supplementary Figures



## References

[1] Kozopas KM, Yang T, Buchan HL, Zhou P, Craig RW (1993). MCL1, a gene expressed in programmed myeloid cell differentiation, has sequence similarity to BCL2. Proc.Natl.Acad.Sci.U.S.A.

[2] Day CL, Chen L, Richardson SJ, Harrison PJ, Huang DCS, Hinds MG (2005). Solution Structure of Prosurvival Mcl-1 and Characterization of Its Binding by Proapoptotic BH3-only Ligands. Journal of Biological Chemistry.

[3] Clohessy JG, Zhuang J, Brady HJM (2004). Characterisation of Mcl-1 cleavage during apoptosis of haematopoietic cells. British Journal of Haematology.

[4] Domina AM, Smith JH, Craig RW (2000). Myeloid Cell Leukemia 1 Is Phosphorylated through Two Distinct Pathways, One Associated with Extracellular Signal-regulated Kinase Activation and the Other with G2/M Accumulation or Protein Phosphatase 1/2A Inhibition. Journal of Biological Chemistry.

[5] Domina AM, Vrana JA, Gregory MA, Hann SR, Craig RW (2004). MCL1 is phosphorylated in the PEST region and stabilized upon ERK activation in viable cells, and at additional sites with cytotoxic okadaic acid or taxol. Oncogene.

[6] Zhong Q, Gao W, Du F, Wang X (2005). Mule/ARF-BP1, a BH3-only E3 ubiquitin ligase, catalyzes the polyubiquitination of Mcl-1 and regulates apoptosis. Cell.

[7] Huang HM, Huang CJ, Yen JJ (2000). Mcl-1 is a common target of stem cell factor and interleukin-5 for apoptosis prevention activity via MEK/MAPK and PI-3K/Akt pathways. Blood.

[8] Le Gouill S, Podar K, Amiot M, Hideshima T, Chauhan D, Ishitsuka K, Kumar S, Raje N, Richardson PG, Harousseau JL, Anderson KC (2004). VEGF induces Mcl-1 up-regulation and protects multiple myeloma cells against apoptosis. Blood.

[9] Wang JM, Chao JR, Chen W, Kuo ML, Yen JJ, Yang-Yen HF (1999). The antiapoptotic gene mcl-1 is up-regulated by the phosphatidylinositol 3-kinase/Akt signaling pathway through a transcription factor complex containing CREB. Mol.Cell Biol..

[10] Ding Q, Huo L, Yang JY, Xia W, Wei Y, Liao Y, Chang CJ, Yang Y, Lai CC, Lee DF, Yen CJ, Chen YJR, Hsu JM, Kuo HP, Lin CY, Tsai FJ, Li LY, Tsai CH, Hung MC (2008). Down-regulation of Myeloid Cell Leukemia-1 through Inhibiting Erk/Pin 1 Pathway by Sorafenib Facilitates Chemosensitization in Breast Cancer. Cancer Research.

[11] Inoshita S, Takeda K, Hatai T, Terada Y, Sano M, Hata J, Umezawa A, Ichijo H (2002). Phosphorylation and inactivation of myeloid cell leukemia 1 by JNK in response to oxidative stress. J.Biol Chem..

[12] Morel C, Carlson SM, White FM, Davis RJ (2009). Mcl-1 Integrates the Opposing Actions of Signaling Pathways That Mediate Survival and Apoptosis. Molecular and Cellular Biology.

[13] Maurer U, Charvet C, Wagman AS, Dejardin E, Green DR (2006). Glycogen synthase kinase-3 regulates mitochondrial outer membrane permeabilization and apoptosis by destabilization of MCL-1. Mol.Cell.

[14] Cuconati A, Mukherjee C, Perez D, White E (2003). DNA damage response and MCL-1 destruction initiate apoptosis in adenovirus-infected cells. Genes Dev.

[15] Willis SN, Chen L, Dewson G, Wei A, Naik E, Fletcher JI, Adams JM, Huang DC (2005). Proapoptotic Bak is sequestered by Mcl-1 and Bcl-xL, but not Bcl-2, until displaced by BH3-only proteins. Genes Dev.

[16] Clohessy JG, Zhuang J, de Boer J, Gil-Gomez G, Brady HJ (2006). Mcl-1 interacts with truncated Bid and inhibits its induction of cytochrome c release and its role in receptor-mediated apoptosis. J.Biol.Chem.

[17] Czabotar PE, Lee EF, van Delft MF, Day CL, Smith BJ, Huang DCS, Fairlie WD, Hinds MG, Colman PM (2007). Structural insights into the degradation of Mcl-1 induced by BH3 domains. Proceedings of the National Academy of Sciences.

[18] Gomez-Bougie P, Ménoret E, Juin P, Dousset C, Pellat-Deceunynck C, Amiot M (2011). Noxa controls Mule-dependent Mcl-1 ubiquitination through the regulation of the Mcl-1/USP9X interaction. Biochemical and Biophysical Research Communications.

[19] Gomez-Bougie P, Oliver L, Le Gouill S, Bataille R, Amiot M (2005). Melphalan-induced apoptosis in multiple myeloma cells is associated with a cleavage of Mcl-1 and Bim and a decrease in the Mcl-1/Bim complex. Oncogene.

[20] Wuilleme-Toumi S, Trichet V, Gomez-Bougie P, Gratas C, Bataille R, Amiot M (2007). Reciprocal protection of Mcl-1 and Bim from ubiquitin-proteasome degradation. Biochem.Biophys.Res.Commun.

[21] Herrant M, Jacquel A, Marchetti S, Belhacene N, Colosetti P, Luciano F, Auberger P (2004). Cleavage of Mcl-1 by caspases impaired its ability to counteract Bim-induced apoptosis. Oncogene.

[22] Ding Q, He X, Hsu JM, Xia W, Chen CT, Li LY, Lee DF, Liu JC, Zhong Q, Wang X, Hung MC (2007). Degradation of Mcl-1 by betaTrCP Mediates Glycogen Synthase Kinase 3-Induced Tumor Suppression and Chemosensitization. Molecular and Cellular Biology.

[23] Inuzuka H, Shaik S, Onoyama I, Gao D, Tseng A, Maser RS, Zhai B, Wan L, Gutierrez A, Lau AW, Xiao Y, Christie AL, Aster J, Settleman J, Gygi SP, Kung AL, Look T, Nakayama KI, DePinho RA, Wei W (2011). SCFFBW7 regulates cellular apoptosis by targeting MCL1 for ubiquitylation and destruction. Nature.

[24] Schwickart M, Huang X, Lill JR, Liu J, Ferrando R, French DM, Maecker H, O'Rourke K, Bazan F, Eastham-Anderson J, Yue P, Dornan D, Huang DCS, Dixit VM (2010). Deubiquitinase USP9X stabilizes MCL1 and promotes tumour cell survival. Nature.

[25] Han J, Goldstein LA, Gastman BR, Rabinowich H (2006). Interrelated Roles for Mcl-1 and BIM in Regulation of TRAIL-mediated Mitochondrial Apoptosis. J.Biol Chem..

[26] Weng C, Li Y, Xu D, Shi Y, Tang H (2005). Specific cleavage of Mcl-1 by caspase-3 in tumor necrosis factor-related apoptosis-inducing ligand (TRAIL)-induced apoptosis in Jurkat leukemia T cells. J.Biol Chem..

[27] Bae J, Leo CP, Hsu SY, Hsueh AJW (2000). MCL-1S, a Splicing Variant of the Antiapoptotic BCL-2 Family Member MCL-1, Encodes a Proapoptotic Protein Possessing Only the BH3 Domain. Journal of Biological Chemistry.

[28] Bingle CD, Craig RW, Swales BM, Singleton V, Zhou P, Whyte MKB (2000). Exon Skipping in Mcl-1 Results in a Bcl-2 Homology Domain 3 Only Gene Product That Promotes Cell Death. Journal of Biological Chemistry.

[29] Boise LH, González-García M, Postema CE, Ding L, Lindsten T, Turka LA, Mao X, Nuñez G, Thompson CB (1993). bcl-x, a bcl-2-related gene that functions as a dominant regulator of apoptotic cell death. Cell.

[30] Kim JH, Sim SH, Ha HJ, Ko JJ, Lee K, Bae J (2009). MCL-1ES, a novel variant of MCL-1, associates with MCL-1L and induces mitochondrial cell death. FEBS Letters.

[31] Fujise K, Zhang D, Liu Jl, Yeh ETH (2000). Regulation of Apoptosis and Cell Cycle Progression by MCL1. Journal of Biological Chemistry.

[32] Jamil S, Sobouti R, Hojabrpour P, Raj M, Kast J, Duronio V (2005). A proteolytic fragment of Mcl-1 exhibits nuclear localization and regulates cell growth by interaction with Cdk1. Biochem.J.

[33] Thomas LW, Lam C, Edwards SW (2010). Mcl-1; the molecular regulation of protein function. FEBS Letters.

[34] Hagenbuchner J, Ausserlechner MJ, Porto V, David R, Meister B, Bodner M, Villunger A, Geiger K, Obexer P (2010). The anti-apoptotic protein BCL2L1/Bcl-xL is neutralized by pro-apoptotic PMAIP1/Noxa in neuroblastoma, thereby determining bortezomib sensitivity independent of prosurvival MCL1 expression. J.Biol.Chem.

[35] Warr MR, Acoca S, Liu Z, Germain M, Watson M, Blanchette M, Wing SS, Shore GC (2005). BH3-ligand regulates access of MCL-1 to its E3 ligase. FEBS Lett.

[36] Mei Y, Du W, Yang Y, Wu M (2005). Puma(*)Mcl-1 interaction is not sufficient to prevent rapid degradation of Mcl-1. Oncogene.

[37] Thomas LW, Lam C, Clark RE, White MRH, Spiller DG, Moots RJ, Edwards SW (2012). Serine 162, an Essential Residue for the Mitochondrial Localization, Stability and Anti-Apoptotic Function of Mcl-1. PLoS One.

[38] Obexer P, Geiger K, Ambros PF, Meister B, Ausserlechner MJ (2007). FKHRL1-mediated expression of Noxa and Bim induces apoptosis via the mitochondria in neuroblastoma cells. Cell Death Differ.

[39] Weng C, Li Y, Xu D, Shi Y, Tang H (2005). Specific cleavage of Mcl-1 by caspase-3 in tumor necrosis factor-related apoptosis-inducing ligand (TRAIL)-induced apoptosis in Jurkat leukemia T cells. J.Biol.Chem.

[40] Geiger K, Hagenbuchner J, Rupp M, Fiegl H, Sergi C, Meister B, Kiechl-Kohlendorfer U, Muller T, Ausserlechner MJ, Obexer P (2012). FOXO3/FKHRL1 is activated by 5-aza-2-deoxycytidine and induces silenced caspase-8 in neuroblastoma. Mol.Biol.Cell.

[41] Ausserlechner MJ, Salvador C, Deutschmann A, Bodner M, Viola G, Bortolozzi R, Basso G, Hagenbuchner J, Obexer P (2013). Therapy-resistant acute lymphoblastic leukemia (ALL) cells inactivate FOXO3 to escape apoptosis induction by TRAIL and Noxa. Oncotarget.

[42] Grignani F, Kinsella T, Mencarelli A, Valtieri M, Riganelli D, Grignani F, Lanfrancone L, Peschle C, Nolan GP, Pelicci PG (1998). High-efficiency gene transfer and selection of human hematopoietic progenitor cells with a hybrid EBV/retroviral vector expressing the green fluorescence protein. Cancer Res.

[43] Ausserlechner MJ, Obexer P, Deutschmann A, Geiger K, Kofler R (2006). A retroviral expression system based on tetracycline-regulated tricistronic transactivator/repressor vectors for functional analyses of anti-proliferative and toxic genes. Mol.Cancer Ther..

[44] Hagenbuchner J, Kuznetsov A, Hermann M, Hausott B, Obexer P, Ausserlechner MJ (2012). FOXO3-induced reactive oxygen species are regulated by BCL2L11 (Bim) and SESN3. J.Cell Sci..

[45] Obexer P, Hagenbuchner J, Rupp M, Salvador C, Holzner M, Deutsch M, Porto V, Kofler R, Unterkircher T, Ausserlechner MJ (2009). p16INK4A sensitizes human leukemia cells to FAS- and glucocorticoid-induced apoptosis via induction of BBC3/Puma and repression of MCL1 and BCL2. J.Biol.Chem.

[46] Hagenbuchner J, Kuznetsov AV, Obexer P, Ausserlechner MJ (2012). BIRC5/Survivin enhances aerobic glycolysis and drug resistance by altered regulation of the mitochondrial fusion/fission machinery. Oncogene.

