# Supplementary material for: A novel Mcl1 variant inhibits apoptosis via increased Bim sequestration
Source: Oncotarget. 2013 Jul 15;4(8):1241–52. doi: 10.18632/oncotarget.1147 (PMC3787154; doi:10.18632/oncotarget.1147)
Supplement: Supplementary file 1 [file oncotarget-04-1241-s001.ppt]

## Slide 1
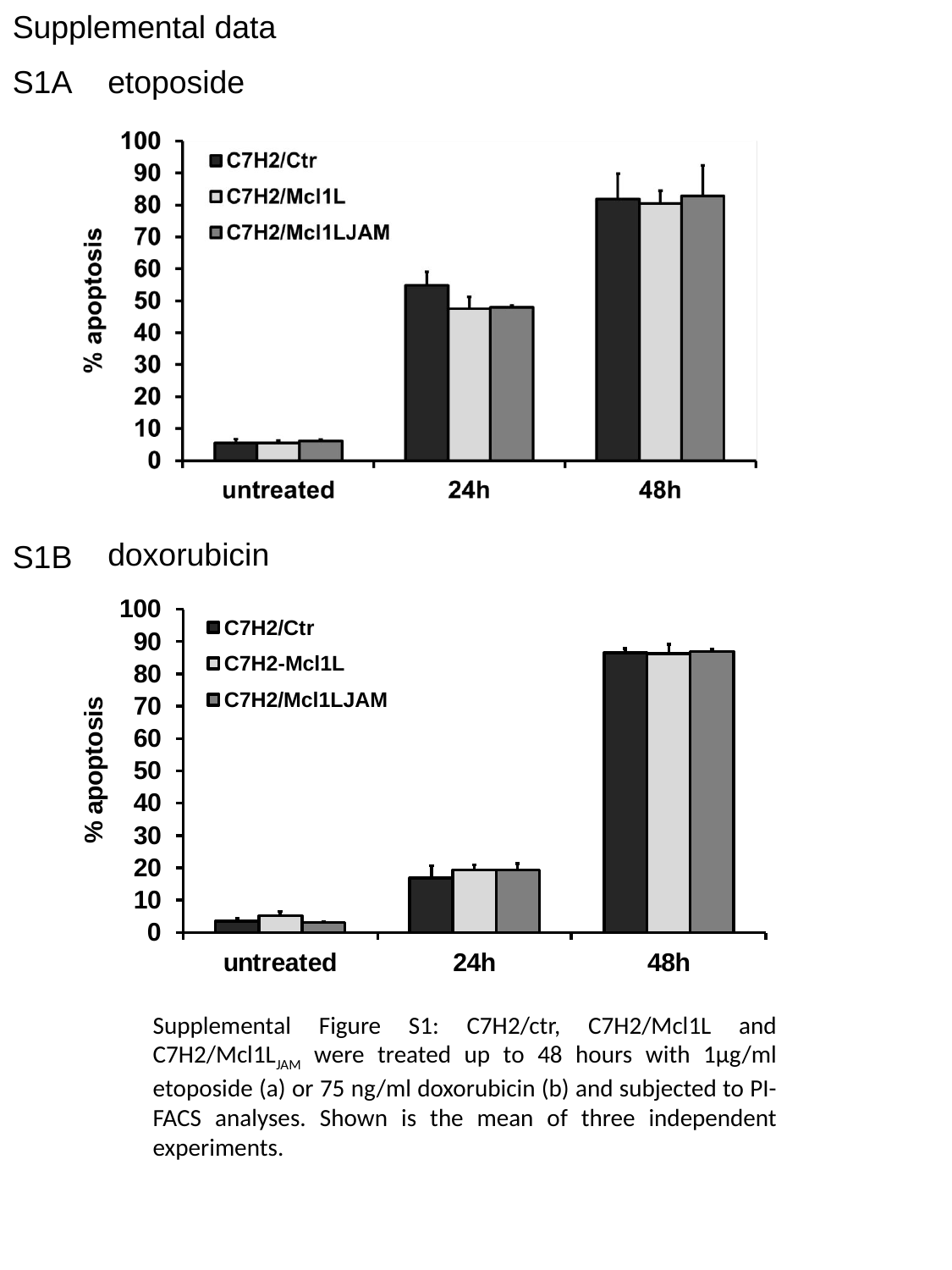

Supplemental data
S1A
etoposide
doxorubicin
S1B
Supplemental Figure S1: C7H2/ctr, C7H2/Mcl1L and C7H2/Mcl1LJAM were treated up to 48 hours with 1µg/ml etoposide (a) or 75 ng/ml doxorubicin (b) and subjected to PI-FACS analyses. Shown is the mean of three independent experiments.

## Slide 2
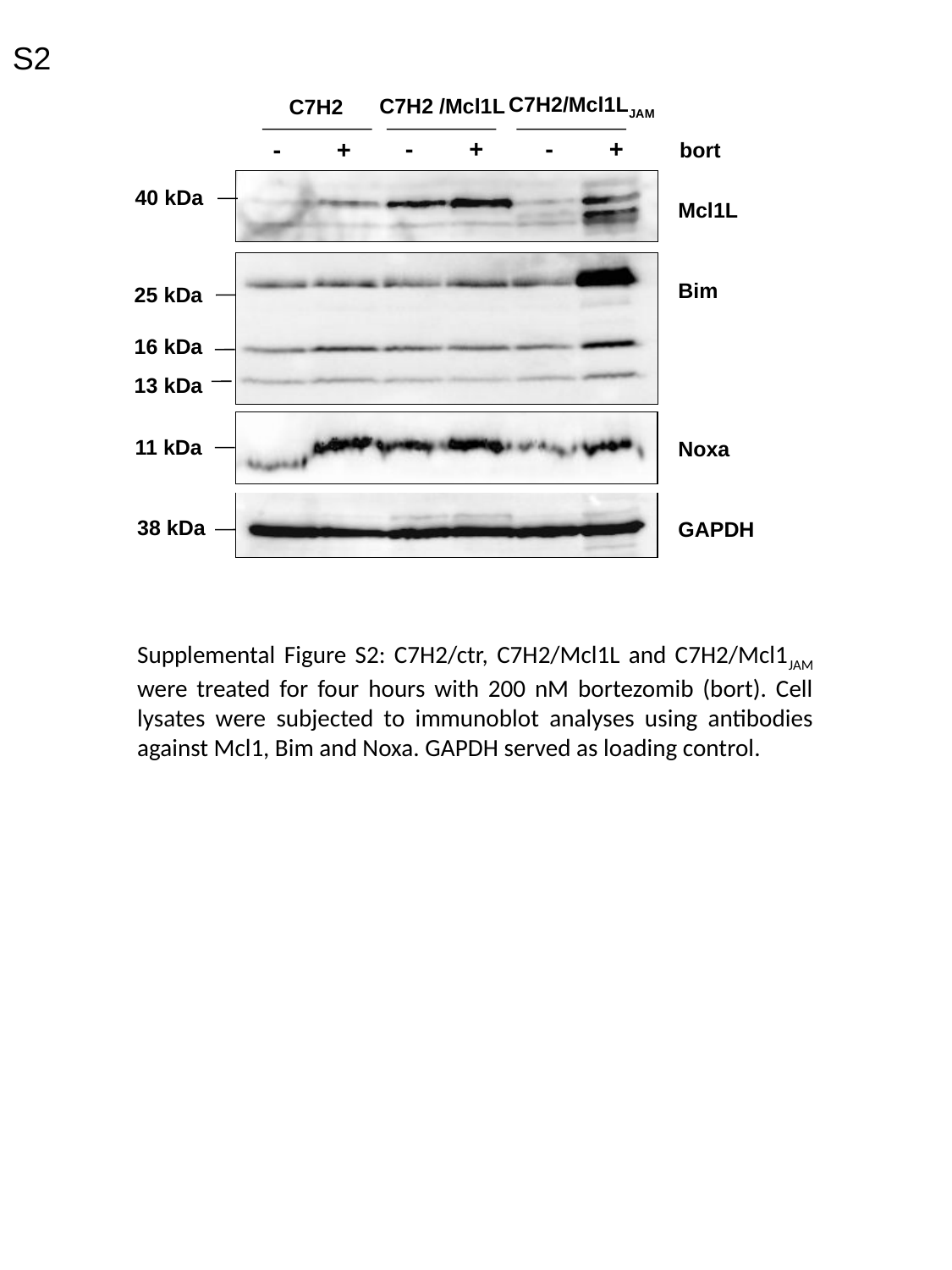

S2
C7H2/Mcl1LJAM
C7H2 /Mcl1L
C7H2
- + bort
- +
- +
40 kDa
Mcl1L
Bim
25 kDa
16 kDa
13 kDa
11 kDa
Noxa
38 kDa
GAPDH
Supplemental Figure S2: C7H2/ctr, C7H2/Mcl1L and C7H2/Mcl1JAM were treated for four hours with 200 nM bortezomib (bort). Cell lysates were subjected to immunoblot analyses using antibodies against Mcl1, Bim and Noxa. GAPDH served as loading control.

## Slide 3
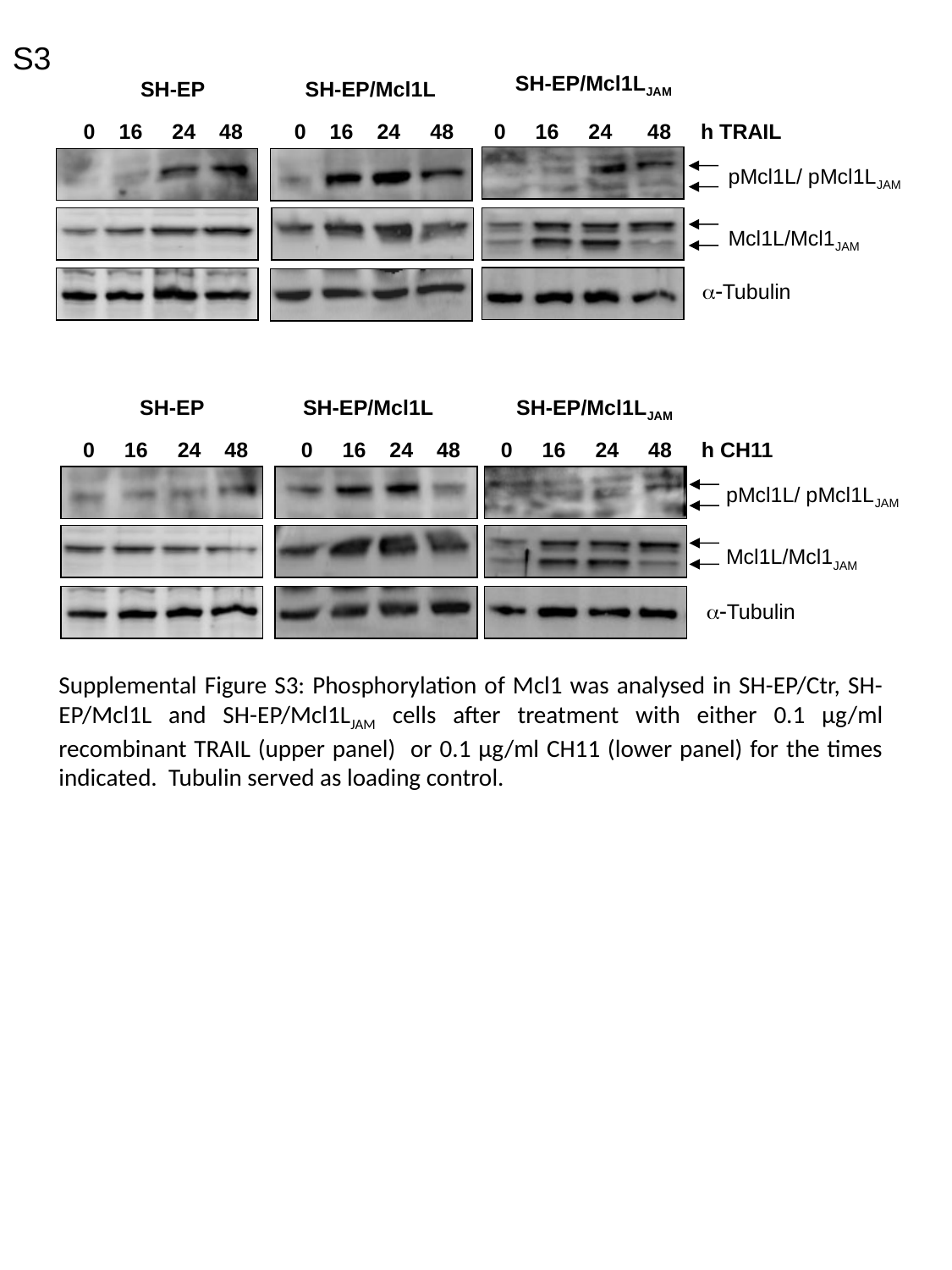

S3
SH-EP/Mcl1LJAM
SH-EP
SH-EP/Mcl1L
0 16 24 48
0 16 24 48
0 16 24 48 h TRAIL
pMcl1L/ pMcl1LJAM
Mcl1L/Mcl1JAM
α−Tubulin
SH-EP
SH-EP/Mcl1L
SH-EP/Mcl1LJAM
0 16 24 48
0 16 24 48
0 16 24 48 h CH11
pMcl1L/ pMcl1LJAM
Mcl1L/Mcl1JAM
α−Tubulin
Supplemental Figure S3: Phosphorylation of Mcl1 was analysed in SH-EP/Ctr, SH-EP/Mcl1L and SH-EP/Mcl1LJAM cells after treatment with either 0.1 µg/ml recombinant TRAIL (upper panel) or 0.1 µg/ml CH11 (lower panel) for the times indicated. Tubulin served as loading control.

## Slide 4
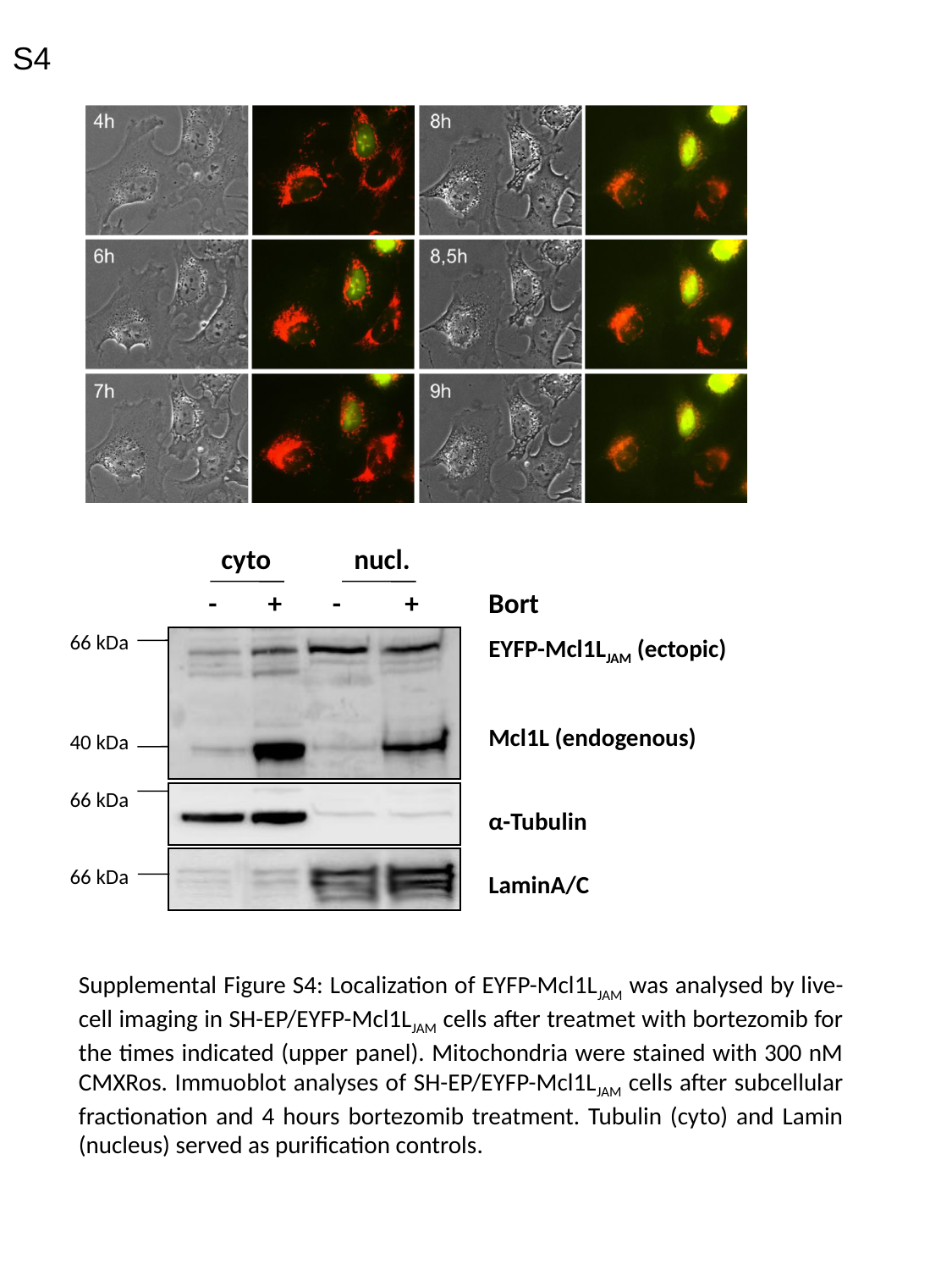

S4
cyto
nucl.
 - + - + Bort
66 kDa
40 kDa
66 kDa
66 kDa
EYFP-Mcl1LJAM (ectopic)
Mcl1L (endogenous)
α-Tubulin
LaminA/C
Supplemental Figure S4: Localization of EYFP-Mcl1LJAM was analysed by live-cell imaging in SH-EP/EYFP-Mcl1LJAM cells after treatmet with bortezomib for the times indicated (upper panel). Mitochondria were stained with 300 nM CMXRos. Immuoblot analyses of SH-EP/EYFP-Mcl1LJAM cells after subcellular fractionation and 4 hours bortezomib treatment. Tubulin (cyto) and Lamin (nucleus) served as purification controls.
